# The role of clinic-based breastfeeding peer counseling on breastfeeding rates among low-income patients

**DOI:** 10.1186/s12884-024-06395-1

**Published:** 2024-04-25

**Authors:** Yetunde Awosemusi, Lauren Keenan-Devlin, Noelle Griffin Martinez, Lynn M. Yee, Ann E. B. Borders

**Affiliations:** 1 Women’s Health of Las Colinas, Medical City Healthcare, 6750 N MacArthur Blvd, Suite 100, Irving, TX 75039 USA; 2 Department of Obstetrics and Gynecology, NorthShore University HealthSystem, 2650 Ridge Ave, Walgreen Building Suite 1507, Evanston, IL 60201 USA; 3 Department of Obstetrics and Gynecology, University of Chicago Pritzker School of Medicine, Chicago, IL USA; 4 Department of Family and Community Medicine, University of California San Francisco, 995 Potrero Avenue Ward 83, San Francisco, CA 94110 USA; 5 Department of Obstetrics and Gynecology, Northwestern University Feinberg School of Medicine, 250 E. Superior St, Suite 5-2145, Chicago, IL 60611 USA

**Keywords:** Breastfeeding, Breastfeeding peer counselor, Initiation, Exclusive, Health disparities, Low-income

## Abstract

**Background:**

Despite the benefits of breastfeeding (BF), rates remain lower than public health targets, particularly among low-income Black populations. Community-based breastfeeding peer counselor (BPC) programs have been shown to increase BF. We sought to examine whether implementation of a BPC program in an obstetric clinical setting serving low-income patients was associated with improved BF initiation and exclusivity.

**Methods:**

This is a quasi-experimental time series study of pregnant and postpartum patients receiving care before and after implementation of a BPC program in a teaching hospital affiliated prenatal clinic. The role of the BPC staff included BF classes, prenatal counseling and postnatal support, including in-hospital assistance and phone triage after discharge. Records were reviewed at each of 3 time points: immediately before the hire of the BPC staff (2008), 1-year post-implementation (2009), and 5 years post-implementation (2014). The primary outcomes were rates of breastfeeding initiation and exclusivity prior to hospital discharge, secondary outcomes included whether infants received all or mostly breastmilk during inpatient admission and by 6 weeks post-delivery. Bivariable and multivariable analyses were utilized as appropriate.

**Results:**

Of 302 patients included, 52.3% identified as non-Hispanic Black and 99% had Medicaid-funded prenatal care. While there was no improvement in rates of BF initiation, exclusive BF during the postpartum hospitalization improved during the 3 distinct time points examined, increasing from 13.7% in 2008 to 32% in 2014 (2009 aOR 2.48, 95%CI 1.13–5.43; 2014 aOR 1.82, 95%CI 1.24–2.65). This finding was driven by improved exclusive BF for patients who identified as Black (9.4% in 2008, 22.9% in 2009, and 37.9% in 2014, *p* = 0.01).

**Conclusion:**

Inpatient BF exclusivity significantly increased with the tenure of a BPC program in a low-income clinical setting. These findings demonstrate that a BPC program can be a particularly effective method to address BF disparities among low-income Black populations.

## Introduction

Breastfeeding has long been recognized to have multiple short- and long-term benefits for both parents and babies. Parents who breastfeed decrease their risk of developing hypertension, type 2 diabetes, breast and ovarian cancer, and their breastfed children have lower rates of infection, asthma, sudden infant death syndrome (SIDS), and are less likely to be overweight or develop diabetes later in life [1, 2]. Given the importance of breastfeeding, several professional and non-governmental organizations, including the World Health Organization (WHO), recommend that all infants should be exclusively breastfed for the first six months of life, with continued breastfeeding up to 2 years of age. Yet rates of breastfeeding remain lower than these recommendations, particularly among low-income parents who identify as Black. In the Centers for Disease Control and Prevention (CDC) National Immunization Survey from 2020, 77.3% of Black parents ever breastfed, compared to rates of 81–87% for parents of other races, and exclusive breastfeeding through 3 months was 39.2% for Black dyads compared to 43.0-49.1% for dyads of other races [3]. Common reasons reported for not initiating breastfeeding and early cessation include infant-specific concerns (difficulty latching, concerns about weight and inadequate milk intake), breastfeeding challenges, cultural norm, lack of support from friends/family, return to school/work, chronic medical problems and medication use while breastfeeding [4–8]. These issues appear to be more prevalent among Black individuals and likely contribute to the lower rates of breastfeeding [9].

Breastfeeding Peer Counseling (BPC) has been found to improve breastfeeding outcomes for low-income communities and families of color. A Breastfeeding Peer Counselor is a lay health worker with similar cultural, demographic, and socioeconomic background to the population they serve, who has had personal success breastfeeding, and has completed a breastfeeding training program [10]. BPC programs have been implemented all over the United States in a variety of practice patterns including the Special Supplemental Nutrition Program for Women, Infants and Children (WIC) programs with most reporting improvement in breastfeeding initiation, exclusivity, and continuation [11–14].

Although there is substantial existing evidence for the effectiveness of BPC as a lactation support strategy in clinical settings [11, 15–21], none of the intervention models published to date included prenatal in-clinic visits with patients, but rather employed home visits [15, 17, 20] or phone encounters [17, 18, 21, 22] for outpatient prenatal education. Five of the existing trials involved BPCs completing inpatient encounters during the delivery admission [11, 17, 19–21], and two utilized outpatient in-clinic visits [11, 18]. A model including BPC encounters during prenatal care, delivery admission, and the postpartum period has not been tested, though an RCT of this model is currently underway [23]. Therefore, the objective of our study was to evaluate whether a clinically-integrated BPC program providing continuity lactation care across the prenatal, inpatient, and postpartum periods improved breastfeeding initiation and exclusivity for all clinic patients, and for patients of color in particular.

## Methods

This was a quasi-experimental time series analysis performed at a hospital-based clinic affiliated with Northwestern Prentice Hospital, a high-volume academic tertiary care center in in Chicago, Illinois delivering approximately 12,000 patients per year.

### Site

The BPC program was based at the Prenatal Ambulatory Care (PAC) clinic, an outpatient teaching practice that serves 300–400 patients with publicly funded prenatal care each year. At this outpatient practice, obstetrics and gynecology residents, maternal-fetal medicine fellows, and faculty provide care for a diverse patient population.

### Peer counselor recruitment, training, and protocol

A 1 FTE BPC staff member was recruited to serve the patient population receiving care at this site. The BPC staff was recruited and hired in coordination with HealthConnect One, a community organization that trains and supports Breastfeeding Peer Counselors. An important element of the BPC protocol is to hire BPC staff from the geographic and ethnic community served, optimizing cultural and racial concordance. Two individuals served in the role over the included time period, the first from 2009 to 2010, and the second from 2010 to 2014. Both BPC staff self-identified as Black, concordant with the majority of patients served in the PAC clinic. The first BPC staff hired had been a prior patient in the PAC clinic, and both BPC staff that served in the role were from communities served by the PAC clinic. The BPC staff was trained to provide both in-person and virtual (telephone) lactation education before delivery, lactation care during the postpartum hospitalization, and anticipatory guidance and lactation trouble-shooting in the outpatient postpartum period. The BPC documented encounters in the electronic medical record.

The BPC staff had an automatically-scheduled 20 min scheduled appointment with all patients during their first or second prenatal visit. During the BPC prenatal visit, newborn feeding options and the benefits of breastfeeding were discussed, and educational material was provided. In addition, the BPC staff helped the patient sign up for prenatal classes, including a breastfeeding class taught by the BPC staff. The BPC breastfeeding class was held monthly, during a clinic day over the lunch hour, with free lunch provided and support persons were encouraged to attend with the patient. The BPC staff also helped arrange breast pump procurement as needed. After delivery, the BPC staff person met with patients to offer breastfeeding support prior to discharge from the hospital. At discharge, all patients received contact information for the BPC warmline to call with breastfeeding questions or concerns. The BPC staff followed up with patients in person at their postpartum visit and via phone. The BPC staff completed prenatal and postpartum clinic visits in standard time blocks, and then completed hospital inpatient postpartum visits in alternate time blocks in order to see all patients receiving care that day. The BPC staff was managed by the PAC Clinic nurse manager and was supported by a physician champion, outpatient prenatal nurse champion, postpartum nurse champion, and lactation consultant champion who met monthly to troubleshoot issues.

### Data collection

This analysis includes electronic medical record (EMR) data from the first 100 delivering patients for each of 3 years: 2008, immediately before the hire of the BPC; 2009, one-year post-implementation of the BPC program; and 2014, five years post-implementation. Data from each period were audited retrospectively while implementation was ongoing. Inpatient breastfeeding data were extracted from feeding flowsheets, and postpartum breastfeeding was identified in the free text charting notes from the postpartum outpatient encounter. Eligible patients were those who received prenatal care at the PAC clinic and delivered a live child at the hospital. For each of these one-year time periods, the participants represented approximately one-third of patients receiving care through the hospital-based outpatient teaching practice. Because there was no EMR documentation of BPC appointments during the 2009 implementation year, exposure to BPC was defined by the period of implementation, either no exposure to BPC during the 2008 baseline period or exposure to BPC as standard care in 2009 or 2014. Aside from the BPC program, there were no significant changes to either outpatient lactation education, nor to the inpatient lactation program, during this period 2008–2014.

Demographic characteristics were collected for the deliveries including age, self-identified race/ethnicity, marital status, and health insurance status. Additionally, we documented whether the infant was born preterm (< 37 weeks of gestation), the route of delivery (cesarean vs. vaginal), and whether the neonate was admitted to the Neonatal Intensive Care Unit (NICU), as these factors may represent significant challenges to breastfeeding success.

The primary outcomes were breastfeeding initiation, defined as at least 1 prolonged feed during the inpatient admission, and breastfeeding exclusivity, defined as only breastmilk feeds during the inpatient admission. Secondary outcomes included whether infants received all or mostly breastmilk during inpatient admission and by 6 weeks post-delivery, defined as 50–100% of feeds composed of breastmilk only. Data were obtained from the inpatient and outpatient medical records via documentation of routine clinical care; no direct patient queries were performed for the purposes of this analysis.

### Statistical methods

To examine the association of the BPC program with breastfeeding support and breastfeeding outcomes, we compared baseline data to each of the two post-implementation time periods. Differences in prenatal breastfeeding support, breastfeeding initiation, breastfeeding exclusivity, mostly breastfeeding during the inpatient admission, and mostly breastfeeding at 6 weeks post-delivery for each of the 2 post-implementation time points were compared to baseline using chi-squared analyses. We also included descriptive data and chi-squared results for the association between the number of consults in the 2014 assessment year. Differences in breastfeeding outcomes from baseline and post-implementation were compared for the full sample and stratified by race/ethnicity using univariate and multivariate logistic regression. Covariates included factors known to be associated with breastfeeding outcomes and included maternal age, race/ethnicity, marital status, route of delivery, and NICU admission for the neonate. All analyses were completed with IBM SPSS v22 [27]. This research was reviewed and approved by the Northwestern University Institutional Review Board (STU00058598).

### Power

US based studies of BPC programs have reported enrollment between 182 and 250 participants and have demonstrated significant improvement in breastfeeding initiation, intensity, and duration between usual care and treatment groups [24], including a recent publication by our team with *n* = 428 that identified significant differences in breastfeeding outcomes between baseline (*n* = 147) and treatment (*n* = 281) within racial and ethnic groups. The cohorts of Black patients were *n* = 45 at baseline and *n* = 56 in treatment [25]. With *n* = 300 our project was adequately powered to detect differences between the baseline and treatment groups overall, and with *n* = 58, *n* = 53, and *n* = 46 Black patients in each cohort we were also powered to detect differences within racial and ethnic groups.

## Results

### Demographics

A total of 302 patients were included in this analysis out of which 102 were in the baseline group, 100 in the one-year follow-up group, and 100 in the 5-year follow-up group. Of these patients, 52.5% identified as non-Hispanic Black and 99% received Medicaid-funded prenatal care (Table 1). Patients from the 3 time points were comparable with regards to race/ethnicity, health insurance, parity, route of delivery, and NICU admission. Patients included in the 2014 assessment were older compared to those included in the 2008 and 2009 assessments. Patients included in the 2014 group were significantly more likely to be married compared to the other groups (35% in 2014 compared to 19.6% in 2008 and 18% in 2009, *p* = 0.019). 70% of patients returned for their 6-week visit and had feeding documented. For the only year the data were available in 2014, 70% of patients had at least 1 BPC consult prenatally with a range of 0–2 consults between 6-weeks of gestation and 6-weeks post-delivery.

**Table 1:** Patient characteristics from BPC program at baseline and follow up

	Baseline 2008 (*n* = 102)		Follow up 2009 (*n* = 100)		Follow up 2014 (*n* = 100)		Differences between groups
Characteristic	*N*	%	*N*	%	*N*	%	*p*
Race/Ethnicity							0.25
White	9	8.8%	15	15.0%	9	9.0%	
Black	58	56.9%	53	53.0%	46	46.0%	
Hispanic/Latine	30	29.4%	26	26.0%	37	37.0%	
Asian	2	2.0%	5	5.0%	4	4.0%	
Other	3	2.9%	1	1.0%	2	2.0%	
Age							< 0.05
18–19	13	12.7%	18	18.0%	8	8.0%	
20–30	67	65.7%	63	63.0%	49	49.0%	
31–40	22	21.6%	19	19.0%	42	42.0%	
41+	0	0.0%	0	0.0%	1	1.0%	
Marital status							0.019
Single	82	80.4%	80	80.0%	61	61.0%	
Married	20	19.6%	18	18.0%	35	35.0%	
Divorced	0	0.0%	2	2.0%	0	0.0%	
Health insurance status							0.85
Self	2	2.0%	0	0.0%	0	0.0%	
Public	97	95.1%	99	99.0%	100	100.0%	
Private	3	2.9%	1	1.0%	0	0.0%	
Parity							0.12
Nulliparous	37	36.3%	45	45.0%	31	31.0%	
Route of delivery							0.31
Cesarean	22	21.6%	26	26.0%	31	31.0%	
NICU admission for infant							0.20
Yes	16	15.7%	10	10.0%	8	8.0%	
Completed 6-week visit							0.27
Yes	71	69.6%	76	76.0%	66	66.0%	
Number BPC consults documented							N/A
Mean prenatal consults	-	-	-	-	0.87 ± 0.7	-	
Mean post-delivery consults	-	-	-	-	0.06 ± 0.2	-	
Mean total consults	-	-	-	-	0.93 ± 0.7	-	

* indicates *p* < 0.05

## Breastfeeding initiation and exclusivity

Exclusive breastfeeding during admission increased significantly across the three time points, from 13.7% in 2008 to 24% in 2009, to 32% in 2014 (Table 2). Improvement in exclusive breastfeeding after BPC program implementation was significant even after adjustment for age, race, marital status, route of delivery, and NICU admission (Table 3: 2009 aOR 2.48, 95%CI 1.13, 5.43; 2014 aOR 1.82, 95%CI 1.24, 2.66). We did not observe significant differences in breastfeeding initiation during admission or breastmilk feeding at 6 weeks post-delivery (Table 2).

For the year the data were available (2014), we examined the association between the number of BPC consults and breastfeeding outcomes in Table 3. Additional BPC visits were not significantly associated with increased breastmilk initiation or exclusivity at any of the time points. There was a trend toward increased proportion of breastmilk feeds in the hospital with more BPC consults.

**Table 2:** Breastfeeding initiation and exclusivity at 5 years’ post-implementation (2014) by number of BPC consults

	No BPC consults *N* = 31	1 BPC consult *N* = 45	2 BPC consults *N* = 24	*p*
Initiation of Breastfeeding	24 (77.4)	33 (73.3)	18 (75.0)	0.92
Exclusive Breastfeeding during admission	8 (25.8)	17 (37.8)	7 (29.2)	0.52
All or mostly breastmilk during inpatient admission	14 (45.2)	27 (60.0)	16 (66.7)	0.24
All or mostly breastmilk at 6 weeks post-delivery	7 (58.3)^a^	10 (34.5) ^b^	8 (66.7) ^c^	0.12

^a^*n* = 12; ^b^*n* = 29; ^c^*n* = 12

To examine the association of the program with breastfeeding disparities, we compared rates of initiation, exclusivity, and duration between racial and ethnic groups during each of the assessment periods (Table 3). Results indicated that the increase in exclusive breastfeeding for the total sample was driven by improved exclusive breastfeeding for Black parents (9.4% in 2008, compared to 37.9% in 2014, *p* = 0.01), which increased the proportion of Black parents providing mostly breastmilk feedings across the assessment period (30.2% in 2008, compared to 54.3% in 2014, *p* = 0.02). Table 4 depicts regression results for breastfeeding outcomes. Patients who received care at the clinic in the first year post-implementation were more than 2 times as likely as those who received care before the BPC implementation to exclusively breastfeed during their delivery admission (aOR = 2.48, 95% CI 1.13, 5.43), and these findings were also observed in the 5-year post-implementation period (aOR = 1.82., 95% CI 1.24, 2.66).

**Table 3:** Breastfeeding outcomes at baseline (2008), 1-year post-implementation (2009), and 5 years post-implementation (2014), overall and stratified by race/ethnicity

	Initiation of Breastfeeding *N* = 302				Exclusive Breastfeeding during admission *N* = 302				All or mostly breastmilk during inpatient admission *N* = 302				All or mostly breastmilk at 6 weeks post-delivery *N* = 213			
	*n* (%)	*n* (%)	*n* (%)	*P*	*n* (%)	*n* (%)	*n* (%)	*P*	*n* (%)	*n* (%)	*n* (%)	*p*	*n* (%)	*n* (%)	*n* (%)	*p*
	2008	2009	2014		**2008**	2009	2014		**2008**	2009	2014		2008	2009	2014	
**Overall**	74 (72.5)	61 (61)	75 (75)	0.32	14 (13.7)	24 (24)	32 (32)	0.02	40 (39.2)	52 (52)	57 (57)	0.04	23 (22.5)	28 (28)	25 (25)	0.68
**White**	9 (100)	9 (64.3)	5 (55.6)	0.08	1 (11.1)	4 (28.6)	3 (33.3)	0.51	7 (77.8)	9 (64.3)	5 (55.6)	0.61	5 (62.5)	4 (50.0)	2 (40.0)	0.72
**Black**	39 (73.6)	31 (64.4)	32 (69.6)	0.62	5 (9.4)	11 (22.9)	17 (37.9)	0.01	16 (30.2)	26 (54.2)	25 (54.3)	0.02	11 (33.3)	14 (37.8)	11 (40.7)	0.83
**Hispanic/ Latine**	21 (72.4)	17 (73.9)	30 (81.1)	0.68	6 (20.7)	7 (30.4)	10 (27.0)	0.71	14 (48.3)	14 (60.9)	22 (59.5)	0.58	6 (37.5)	6 (37.5)	8 (50.0)	0.71
**Asian**	2 (100)	3 (75)	4 (100)	0.44	0 (0.0)	1 (25)	2 (50)	0.46	0 (0.0)	2 (50.0)	4 (100)	0.05	0 (0.0)	3 (75.0)	3 (100)	0.14
**Other**	3 (100)	1 (100)	2 (100)	n/a	2 (66.7)	1 (100)	0 (0.0)	0.19	3 (100)	1 (100)	1 (50.0)	0.30	1 (100)	1 (100)	1 (100)	n/a

**Table 4:** Adjusted odds of breastfeeding outcomes at 2009 and 2014 post-implementation assessment time points compared to 2008 baseline

	2009 Assessment aOR, 95% CI	*P*	2014 Assessment aOR, 95% CI	*p*
Breastfeeding initiation	0.59 0.29, 1.18	0.14	0.79 0.55, 1.16	0.23
Exclusive breastfeeding during inpatient admission	2.48 1.13, 5.43	0.02	1.82 1.24, 2.66	< 0.01
All or mostly breastmilk during inpatient admission	1.98 1.07, 3.69	0.03	1.38 1.00, 1.89	0.05
All or mostly breastfeeding at 6 weeks post-delivery	1.08 0.51, 2.30	0.84	1.03 0.66, 1.59	0.91

*adjusted for age, race and ethnicity, marital status, route of delivery, and NICU admission for the neonate

## Discussion

### Principal findings

The implementation of a BPC program in a clinic serving low-income patients was associated with higher rates of exclusive breastfeeding during inpatient admission. The increase in exclusive breastfeeding persisted even after adjusting for factors commonly known to be associated with breastfeeding, including age, race, marital status, route of delivery, and NICU admission. Importantly, the increase in breastfeeding exclusivity in our study population was primarily driven by improvements in breastfeeding among Black patients, whose exclusive breastfeeding rates increased from 9.4% at baseline in 2008 to 37.9% in 2014. However, the program had no significant association with the initiation of breastfeeding, nor with breastfeeding rates at 6 weeks post-delivery.

### Results

Similar to our study, a 2002 study by Pugh [20] implemented a clinically-integrated Breastfeeding Peer Counselor intervention among a predominantly Black population and found that those who received BPC support were significantly more likely to exclusively breastfeed at 3 months (25% vs. 45%) and 6 months (15% vs. 30%).

Improvement of exclusive breastfeeding rates at discharge demonstrated in our study suggests that implementation of BPC in clinical settings may be a useful strategy for healthcare organizations to increase their exclusive breastfeeding rates, which are one of the five perinatal quality measures assessed by accrediting organizations like the Joint Commission. Our program showed no improvement in breastfeeding initiation associated with the BPC program. This may be due to already high breastfeeding initiation rates of 72.5% in our 2008 baseline, which was higher than the 71.2% of birthing parents in Illinois who initiated breastfeeding in 2008 per the CDC [26]. Another possible explanation is that BPC program may not necessarily change attitudes towards breastfeeding, as most patients at baseline chose to initiate breastfeeding, but rather BPC programs may prioritize helping parents achieve their breastfeeding goal. A study by Srinivas [11] found that although there was no difference in rates of exclusive breastfeeding, breastfeeding initiation, or duration for those randomized to BPC care versus standard lactation care, significant improvement in breastfeeding rates and duration occurred among patients who already had a positive attitude towards breastfeeding.

Despite the increase in the exclusive breastfeeding during admission, by 6 weeks postpartum only about a quarter of babies were receiving all or mostly breastmilk, a number which was stable from 2008 to 2014. These results are low but not surprising given that the focus on our BPC program was primarily antenatal and in hospital care. Similarly, the 2010 Pugh [27] trial identified that, as BPC support reduced in the postpartum period, so too did improved rates of breastfeeding. Collectively, these results indicate the importance of BPC care continuity [28, 29], and highlight a potential limitation of BPC care that is integrated into a perinatal care schedule with limited post-discharge touch points. Moreover, while peer counselors are able to provide modeling, education, support, and individualized teaching and problem-solving, peer support alone may not be sufficient to overcome suboptimal employment conditions, including limited parental leave, as well as lower job control/autonomy, that contribute to Black-white breastfeeding inequities [30–33]. Ultimately, that our program had the greatest impact during the immediate postpartum hospitalization suggests that the intervention was best suited to address skill-based and relational/support needs, but that additional community and policy interventions are necessary to address the structural determinants of breastfeeding inequities.

### Clinical implications

Our findings highlight the potential for clinically integrated BPC to reduce disparities in breastfeeding outcomes, particularly for Black patients who face the most barriers to breastfeeding success. A study by Bartick and colleagues [34] estimated that suboptimal breastfeeding among Black and Hispanic/Latine families contributes to 2–3 times increased morbidity and mortality for Black and Hispanic/Latine infants compared to White infants. This health burden also contributes to economic burden, costing Black families in particular $400 more in medical costs compared to White families [34] and a total health system cost of approximately $28 million [35]. Given the health and economic impact of breastfeeding, achieving equity in breastfeeding rates can decrease morbidity and mortality in the Black population.

There are few published examples of clinically-integrated BPC programs with continuity across the perinatal period [23], and our results suggest that this model may optimize breastmilk feeding during the inpatient stay.

As demonstrated in the study by Bartick [34], non-exclusive breastfeeding is associated with greater burden of necrotizing enterocolitis for preterm infants and twice as many infant deaths for Black families compared to White families. Therefore, the adoption of a BPC program similar to the program evaluated here may help reduce neonatal morbidity and mortality vis a vis more intensive breastmilk feeding in the critical first days after delivery. Additionally, the BPC program model examined here demonstrated sustained impact across the first 5 years of the program, suggesting the feasibility of sustaining BPC in a clinical setting as enhancement to existing lactation support models.

### Research implications

Our study contributes to the limited studies on the use of a clinically integrated BPC with long term outcomes. Large, randomized studies of BPC are needed to assess whether BPC interventions can close the disparities gap.

### Strengths and limitations

Our study leveraged Electronic Medical Record (EMR) data to examine breastfeeding intensity/exclusivity, an end point which is often not examined in other studies and is one our strengths. In addition, the long-term nature of our study with data available 5 years post implementation of the BPC program helps us examine the lasting effect of the program. Finally, ours is one of few studies on the use of a clinically integrated antenatal and postnatal BPC programs.

There are several limitations to note. Though there were no changes to the institutional lactation care protocols during this time period, the pre-post, retrospective design of the study makes is difficult to identify unmeasured confounding factors that may have contributed to changing breastfeeding patterns across the period such as local, state, and nationwide policies, demographic shifts in the clinical population, or shifting cultural attitudes towards breastfeeding. Indeed, during the study period, there were state-wide and national increases in breastfeeding rates, which may have obscured the effects of our BPC intervention specifically. Nonetheless, we note that the degree of improvement in inpatient exclusive breastfeeding for a predominantly Black patient population receiving public health insurance (10% increase between 2008 and 2009, 18% increase from 2008 to 2014) seen in our study to far exceed the 1% and 10% respective increases observed during those same periods for the US population overall [3]. A second limitation is that these data are older, though we believe still relevant given the increasing attention to strategies to reduce disparities in perinatal care and interventions based on community health worker care integration in particular. The cohort was small, and although a representative sample was selected, it is possible we were underpowered to detect differences in all racial and ethnic groups. By using data collected for quality improvement purposes we were limited to gestational age at delivery and NICU status as proxies for maternal and child health status. Similarly, we were limited to breastfeeding at discharge and 6 weeks postpartum as short-term outcomes, although these are known to be suboptimal indicators of long-term breastfeeding outcomes. Lastly, although the study involved a diverse patient population, it is possible that generalizability may be limited given the single study site and a single BPC administering the intervention.

## Conclusion

Our findings suggest that the implementation of a clinically-based BPC program in a low-income clinical setting was associated with significantly increased rates of breastfeeding exclusivity prior to postpartum hospital discharge, particularly among Black patients. Future studies should focus on standardizing the intervention and improving and disseminating the program to see if results can be replicated in different settings and patient populations. In addition, future work could benefit from qualitative studies to better understand how patients responded to the BPC program and what factors were most critical to helping them achieve their breastfeeding goals.

## Data Availability

The datasets generated and/or analyzed during the current study are not publicly available due to the privacy limitations but are available from the corresponding author on reasonable request.

## Abbreviations

BF: Breastfeeding
BPC: Breastfeeding peer counselor
SIDS: Sudden infant death syndrome
WHO: World Health Organization
CDC: Centers for Disease Control and Prevention
WIC: Women, Infants and Children
PAC: Prenatal Ambulatory Care
NICU: Neonatal Intensive Care Unit
EMR: Electronic Medical Record
